# The community mental health team fidelity scale: A measure of program fidelity of social networks interventions for severe mental illness

**DOI:** 10.3389/fpsyg.2023.1076791

**Published:** 2023-02-22

**Authors:** Mauricio Alvarez-Monjaras, Melissa Lotmore, Russell Razzaque, Mark Steven Hopfenbeck, Stephen Pilling

**Affiliations:** ^1^Department of Clinical Educational and Health Psychology, University College London, London, United Kingdom; ^2^North East London NHS Foundation Trust, London, United Kingdom; ^3^Department of Health Sciences, Norwegian University of Science and Technology, Trondheim, Sør-Trøndelag, Norway

**Keywords:** open dialogue, fidelity, implementation science, community mental health, measure development, severe mental illness, complex interventions

## Abstract

Open dialogue (OD) is a multi-component therapeutic and organizational intervention for crisis and continuing community mental health care with a therapeutic focus on clients’ social networks. The development and implementation of this model of care in the United Kingdom requires considerable contextual adaptations which need to be assessed to support effective implementation. Program fidelity–the extent to which core components of an intervention are delivered as intended by an intervention protocol at all levels–is crucial for these adaptations.

**Aims:**

To develop, pilot, and implement a program fidelity measure for community mental health services providing OD and ‘treatment as usual’ (TAU) or standard NHS crisis and community care.

**Methods:**

Measure structure, content, and scoring were developed and refined through an iterative process of discussion between the research team and OD experts. Measure was piloted in the 6 OD and 6 TAU services participating in a large-scale research program.

**Results:**

Initial data suggests that the Community Mental Health Team Fidelity Scale (COM-FIDE) is a potentially reliable and feasible measure of the fidelity of community mental health services and specific OD components of such services.

## Introduction

Poor social networks have been associated with both the development and maintenance of mental illness (Giacco et al., 2012). Interventions targeting social networks–such as the Open Dialogue (OD) approach (Seikkula et al., 1995) might therefore help ameliorate mental health crises and reduce the likelihood of relapse. However, due to limited staff training and skills, and a lack of continuity associated with the current model of crisis and continued community care of the British National Health Service (NHS), such interventions are not currently provided (Razzaque and Wood, 2015; The Commission on Acute Adult Psychiatric Care, 2015). Further, the professional and contextual adaptations required to integrate OD successfully and sustainably into NHS models of care require a consideration of the model’s core components.

Program fidelity or the extent to which core components of an intervention are delivered as intended by a treatment protocol is a useful approach to supporting effective implementation (Santacroce et al., 2004; Borelli, 2011; Gearing et al., 2011). This paper outlines the development, piloting, and implementation of a program fidelity measure for the OD approach: The Community Mental Health Team Fidelity Scale (COM-FIDE). The paper begins with a brief description of Open Dialogue and the current NHS model of crisis and continuing community care in mental health. This is followed by an exploration of some of the challenges involved in integrating OD into the provision of mental health services in the United Kingdom, including the challenges in developing a fidelity measure. The COM-FIDE development and piloting method are then outlined, alongside some preliminary psychometric data. Finally, results are considered alongside the utility of COM-FIDE.

### Crisis and continuing community mental health care in the United Kingdom

The NHS is facing significant problems in providing care and support for people with severe mental illness, potentially due to poorly developed and increasingly fragmented pathways of care (NHS Confederation, 2016; The Kings Fund, 2016). This is in part a consequence of the functional model of mental health care, where care is often provided by several different teams, each with its own criteria for acceptance (Morton and Norman-Nott, 2019). Standard NHS crisis and continuing community care services for people experiencing severe mental illness consist primarily of crisis resolution and home treatment teams (CRTs) and community mental health teams (CMHTs). As an alternative to hospitalization, these multidisciplinary teams–typically conformed by psychiatrists, mental health nurses, social workers, and support workers–provide intensive assessment, care, and support in patients’ homes (Weisman, 1989; Jethwa et al., 2007; Johnson, 2013). Standard care often acknowledges and may attempt to work with the social network of a person in crisis; however, their brief and functional nature and the pressures on service resources make this form of ongoing network-oriented care a challenging endeavor (Razzaque and Wood, 2015).

Despite the promise shown in randomized controlled trials (Johnson et al., 2005a,b; Lloyd-Evans et al., 2014, 2019), questions have been raised on whether standard care might be decreasing in effectiveness (Johnson et al., 2005a,b; Jacobs and Barrenho, 2011). Wheeler et al. (2015) suggested this might be due to a considerable atrophy of its key functions, with many services offering limited home visits outside of office hours and only 50% of services providing post-hospital discharge care. It is important to ask whether this possible decrease in the quality of community-based services can be explained by a lack of resources or if organizational problems, such as staff competencies, roles, care pathways, or fidelity to a model, may also be contributory factors.

### Open Dialogue

Open Dialogue (Seikkula et al., 1995) is both a therapeutic approach and a way of organizing mental health services developed in Finland, which explicitly targets social networks. The aim of Open Dialogue is to promote a greater shared understanding of service users’ problems, a greater sense of agency, collaborative decision-making, and the network’s mutual support in the long term (Seikkula et al., 1995, 2006; Seikkula et al., 2001a, 2011). This is done through the enactment of the principles of (1) immediate help, (2) social networks perspective, (3) flexibility and mobility, (4) responsibility, (5) psychological continuity, (6) tolerance of uncertainty, and (7) dialogue and polyphony (Seikkula et al., 1995). In contrast to current models of care–in which families may not be directly involved–Open Dialogue uses network meetings attended by family members, friends, and other professionals involved with the service user as the central means of intervention delivery (Seikkula et al., 1995; Seikkula and Olson, 2003; Lakeman, 2014; Razzaque and Wood, 2015). Service users and their social networks engage in shared decision-making with healthcare professionals to agree on appropriate pharmaceutical, psychological, or social interventions (Seikkula et al., 2006; Olson et al., 2014).

The development of an integrated OD approach to the provision of mental health services offers the possibility of an alternative to the current ‘functional team’ model of care in the United Kingdom (Hopfenbeck, 2015; Razzaque and Stockmann, 2016). Preliminary evidence suggests that OD may be more effective than standard care in reducing relapse and the use of antipsychotic medication (Seikkula et al., 2001b, 2003; Hartman and De Courcey, 2015; Bergström et al., 2018). Additionally, OD might help equip mental health staff with additional skills necessary to engage service users and their families across the broad spectrum of care needs (Holmesland et al., 2014). However, although promising, there is no high-quality evidence to date to support an NHS-wide adoption of this model of care.

### Program fidelity measurement

Transferring Open Dialogue from one health care setting to another requires considerable contextual adaptations that could undermine structural (i.e., organizational) and process (i.e., therapeutic) components of the original model (Gonzalez Castro et al., 2004). In fact, international OD implementation programs (e.g., Pocobello and Salamina, 2015) have noted that the organizational change is such, that staying faithful to the OD principles (e.g., Seikkula et al., 2006; Olson et al., 2014) has encountered significant obstacles. Program fidelity or the extent to which an intervention is delivered as intended in a treatment protocol at all levels can be a useful tool for understanding an intervention’s critical components on a structural, organizational, and functional level (Carroll et al., 2007; Proctor et al., 2011; Teague et al., 2012).

Literature suggests that program fidelity measures should involve (1) an evidence-based, comprehensive, and multimodal approach to assessment, (2) clearly and objectively operationalized components stemming from a coherent and comprehensive theory of change, and (3) easily-available data from the relevant stakeholders (Schoenwald et al., 2011; Essock et al., 2015). Although uncommon, existing measures for multi-component interventions such as OD are somewhat consistent in terms of measure design, assessment procedures, and scoring. Donabedian (1988) suggested a structure-process-outcome framework for fidelity evaluation; however, most measures emphasize structural features of service provision (e.g., operations, staffing, or services provided) but tend to neglect important process and outcome features relevant to the therapeutic model (Alvarez-Monjaras, 2019).

A few efforts have been made to establish appropriate fidelity measures for standard crisis and continuing community care. The CORE CRT (Lloyd-Evans et al., 2016) is the most robust and validated measure to date for crisis services. However, OD implementation studies so far have focused on practitioner adherence or the quality of delivery of network meetings according to the key OD principles (Eiterå et al., 2014; Olson et al., 2014; Rambøll, 2014; Ziedonis et al., 2018; Lotmore et al., 2022). Since OD is not only a therapeutic model but also a way of organizing care, it is important to identify not only the clinically relevant (i.e., process) features but also the structural and organizational features that characterize the approach and distinguish it from standard care. In other words, if OD is to be successfully implemented and integrated into the traditional NHS model of crisis and continued community care, it is essential to develop a program fidelity measure to support the implementation of OD that is faithful not only to the original Finnish model, but also fit for its incorporation into the NHS.

## Study aims

This study was part of the NIHR ODDESSI (Open Dialogue: Development and Evaluation of a Social Network Intervention for Severe Mental Illness) program grant (RP-PG-0615-20,021). ODDESSI aims to evaluate whether OD –when integrated within standard NHS mental health services for adults in crisis–improves the clinical and cost-effectiveness of standard crisis and continuing community mental health care (i.e., CRTs and CMHTs). The ODDESSI is a cluster-randomized controlled trial (RCT) consisting of five work packages oriented toward defining, implementing, and evaluating OD services across 28 trial clusters from five NHS trusts (for full protocol see Pilling et al., 2022).

The key goal of the present study was to develop, pilot, and implement a program fidelity measure that could accurately characterize the quality of both standard NHS crisis and continuing community care (hereafter referred to as ‘treatment as usual’ or TAU) and high-quality OD practice. If successful, this measure would provide information on whether: (1) NHS services, once reorganized on an OD model of care, can deliver OD with sufficient fidelity to its core principles and ensure they are both provided effectively; (2) it is possible to distinguish OD services from standard care based on their model of work; and (3) there are any differences in implementation between each model’s teams.

## Methods

### Study design

Although this specific study was relevant to all work packages of the ODDESSI trial, it was embedded in the second work package as part of the feasibility stage (WP2). WP2 addressed the feasibility of a cluster RCT, including the question of whether adherence and fidelity measures could provide a reliable measure of OD practice. Additionally, the NIHR shared their concern that–in order to draw meaningful conclusions from the outcomes–the trial needed to be able to compare OD teams against high-quality TAU. Developing a program fidelity measure is one way of ensuring high quality of care in both OD and TAU.

### Participants

Participants for this study were staff members from six OD services and their six TAU counterparts. For each service, one pair of managers and one pair of practitioners (i.e., psychiatrists, psychologists, psychotherapists, nurses, social workers, and support workers) were interviewed by two joint independent raters. A total of 48 staff members were interviewed.

### Ethical approval

This study received ethics approval from the Health Research Authority under reference number 18/LO/0026. No personal or confidential information was solicited. Participants gave consent for being recorded using an encrypted and password-protected recorder.

### Measure development

**Figure 1:**
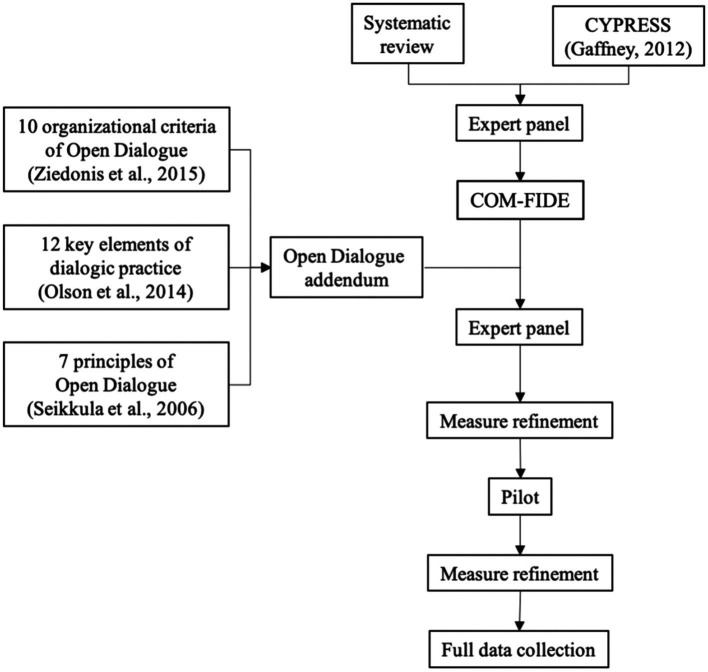
Community Mental Health Team Fidelity Scale (COM-FIDE) development process.

The Community Mental Health Team Fidelity Scale (COM-FIDE) was developed following a stepwise approach (Bond et al., 2000; Holmbeck and Devine, 2009), based on our systematic review of existing measures (Alvarez-Monjaras, 2019), and a series of discussions with experts (Figure 1).

#### Defining the content and scope of the measure

The initial content, method of delivery, and scoring process of the COM-FIDE builds on work done at University College London on the ‘Children and Young People – Resource, Evaluation and Systems Schedule’ (CYPRESS) (Gaffney, 2012) and findings from our systematic review. CYPRESS was developed for the Systemic Therapy for At-Risk Teens (START) RCT (Fonagy et al., 2013) to characterize services delivering multisystemic therapy and management as usual for young people with complex presentations. CYPRESS captures key elements of effective implementation efforts (e.g., coherent theoretical basis, high program fidelity, qualified staff, sustained approach, etc.) across three levels of service delivery: service characteristics, team operations, and delivery of interventions. The promising results from the START trial suggested that CYPRESS could be a robust measure for service characterization.

Drawing on the CYPRESS (Gaffney, 2012), our systematic review, and Donabedian’s (1988) structure-process-outcome framework, the research team agreed to four broad key domains to assess: (1) team structure and culture, (2) access to and engagement with services, (3) delivery of care, and (4) external support. An initial list of items was drafted and then refined based on three factors: (1) a focus on adult mental health, (2) the ability to encompass both OD and TAU, and (3) the ability to identify high-quality TAU.

#### Designing the measure

The refinement and detail of the measure outline was established through a series of meetings and discussions between the main author (MA, Clinical Psychologist), SP (Clinical Psychologist with expertise in evidence-based practice and experience in measure development), RR (Consultant Psychiatrist, ODDESSI co-applicant, and international expert in OD), MH (Lead OD trainer), and ML (Clinical Psychologist involved in the development of the adherence measure). An iterative process, aimed at achieving an acceptable level of utility of the measure, took place between October 2017 and January 2018.

#### Open Dialogue fidelity

Another important goal of WP2 was to refine the OD protocol for its implementation across NHS sites. Consequently, the resulting measure needed to be able to recognize features specific to open dialogue in OD teams. A similar item development process for an OD addendum took place based on existing OD literature (e.g., Seikkula et al., 2006; Olson et al., 2014; Ziedonis et al., 2015), and the ODDESSI treatment protocol. The ODDESSI protocol set out key functions, referral pathways, and governance arrangements of each site, and was developed by the research team in collaboration with experts in OD and TAU, alongside senior NHS staff and clinicians.

Given the complexity of OD terminology, a series of discussions around the main theoretical principles (e.g., dialogism, transparency, openness) were arranged with OD experts. The aim was to determine the best possible way to translate these key principles into objective and reliable service-level items that could be ascertained by raters not trained in OD.

### The community mental health team fidelity scale

The above led to the development of a 25-item Community Mental Health Team Fidelity Scale (COM-FIDE, formerly CoMFideS). COM-FIDE is a measure designed to describe the structure, functioning, pathways, community links, and delivery of care provided by good quality community MH services, including OD. COM-FIDE is hence a measure of program fidelity of both (a) standard NHS crisis and continued community care and (b) best practice in OD delivery. The COM-FIDE also includes a 7-item Open Dialogue Addendum focused on measuring the level of fidelity to open dialogue principles of care.

The first section of the COM-FIDE concerns structural aspects of the services under assessment. The COM-FIDE comprises four sections that assess the level of fidelity of mental health teams–regardless of their model of care–to high-quality crisis and continued community care: (1) Team structure and culture (8 items); (2) Access and engagement (6 items); (3) Delivery of care (6 items); and (4) External and support (5 items).

### Measure refinement

The COM-FIDE was piloted in one OD and one TAU service to identify areas of improvement in the COM-FIDE and assess the measure’s acceptability. For each team, two managers and two practitioners were interviewed using the draft measure, followed by a brief discussion on its structure, content, and acceptability. Using the outcome from the pilot, the measure was once again refined and discussed with the expert panel (See Figure 1).

#### The COM-FIDE manual

Based on initial work gathered throughout the measure development process and drawing from CYPRESS (Gaffney, 2012), a manual was developed (Available on the UCL ODDESSI website). The manual includes a description and rationale for each item alongside their respective behavioral anchors for scoring (Alvarez-Monjaras and Pilling, 2019).

### Measure pilot

#### Recruitment and setting up the interviews

As per the manual, initial contact with services was done *via* email correspondence, describing the study, its purpose, and a brief description of the measure and interview process. Service documentation (e.g., staffing, supervision, safeguarding, and operational policies) was also requested from each Trust to gather service-level data. Interviews typically lasted no more than 60 min. The average time spent per interview was 46 min (range = 35–57). None of the raters were OD-trained but were all clinicians trained on the use of COM-FIDE, which included discussions of each item and rating examples.

#### Agreeing on final ratings

Once each interview session was completed, both raters reviewed their individual score sheets separately. Each item was then jointly reviewed to identify and record disagreements and a consensus reached on the final score.

#### Scoring and cut-off scores

During each interview, both raters simultaneously and independently rate a copy of the COM-FIDE. Once finished, they review and reach a consensus in the ratings. All items of the COM-FIDE are rated on a 4-point behaviorally anchored Likert scale. Advised that a 4-tier structure might offer the highest level of precision possible for rating program fidelity, rather than the traditional 5-point Likert approach. On all items, a score of one indicates that the principle at hand is not present or there is insufficient evidence of its enactment in the team’s way of functioning, whereas a score of 4 indicates that the principle is enacted or carried out in an excellent manner and with no visible shortcomings or inconsistencies across the team. The overall COM-FIDE score yields to a final score of 100 and the OD-addendum to a score of 28. Each section then obtains an average score of its composite items (for more information on scoring and all behavioral anchors, please refer to the manual on the UCL ODDESSI website).

Providing (1) this is the first fidelity measure developed for open dialogue in the NHS, and (2) that there are no pre-existing criteria for what constitutes a ‘good’ standard of TAU care, nor of open dialogue fidelity, we considered 4 fidelity gradations: an average score on each section equal or above 3.40 (85^th^ percentile) was considered ‘very good’; scores between 2.80 and 3.39 (70-85^th^ percentiles) as ‘good’; scores between 2.40 and 2.79 (60-69^th^ percentiles) as ‘acceptable’; and scores equal or below 2.39 (below 60^th^ percentile) as ‘poor’ or lacking fidelity.

### Data analysis

Data from each site consisted of: (1) three rating sheets (i.e., two independent rating sheets and a final rating sheet) for manager interviews, and (2) three rating sheets for practitioner interviews. Data from all rating sheets were entered into an Excel spreadsheet and later exported onto an SPSS database. All analyzes were conducted using IBM SPSS Statistics version 25 for Mac (IBM Corporation, 2017). Descriptive statistics and radar plots were used at service level to characterize site fidelity scores. Statistical tests comparing scores were not conducted given the small sample size.

#### Psychometric properties

The present study explored–albeit tentatively–the following psychometric properties of the COM-FIDE: (1) inter-rater reliability, (2) internal consistency, and (3) face and content validity.

#### Reliability

Reliability analyzes were based on item-level data from the independent rating sheets. In terms of inter-rater reliability, Pearson’s *r* or intra-class coefficients (ICC) were not obtained given the sample size, and that respondents and raters were not fully crossed or nested. Neither of these tests can remove systematic coder deviations and can therefore underestimate the true reliability of ill-structured measurement designs (ISMDs) such as the one used for this study (Putka et al., 2008; Hallgren, 2012). The G estimation coefficient (Putka et al., 2008) was chosen to make up for the limited data and as a less biased reliability estimator. A G coefficient above 0.7 was considered acceptable. Internal consistency reliability was assessed using Cronbach’s alpha (Cronbach, 1951). Alpha coefficients above 0.7 were considered acceptable (Streiner, 2003).

#### Validity

Face and content validity were assumed as adequate given the iterative feedback and input from experts, managers, and staff members. Other forms of measure validity were not considered given the scarcity of data.

## Results

### Service characteristics

All TAU and OD interviews were completed with no missing data. Only TAU teams were able to provide copies of their operational policies as OD teams were still in the process of developing their own; however, given the structure of the trial clusters, TAU policies were also taken to apply to OD teams. The average caseload per staff member was 25.8 service users (SD = 7.36, range = 20–40) for the OD teams and 29.8 (SD = 8.50, range = 25–45) for TAU teams. The mean staff for OD teams was 9.5 (SD = 3.08, range = 5–13) and for TAU teams was 13.8 (SD = 3.49, range = 10–19). Psychiatrists, psychiatric nurses, clinical psychologists, and psychotherapists were the most common professions and were all employed across teams (*n* = 6). Occupational therapists were employed by 83% (*n* = 5) of TAU teams, whereas only in 50% of OD teams. Only one TAU team (8%) employed advocates. Nurse assistants were employed by 25% of the teams (*n* = 3) altogether (Table 1).

**Table 1:** Service characteristics.

	Open Dialogue (*n* = 6)		Standard care (*n* = 6)	
	$\bar{X}$ (Range)		$\bar{X\;}$ (Range)	
Employed staff (FTE and WTE)	9.50 (5–13)		13.82 (10–19)	
Caseload	$\bar{X}\;$ (SD)		$\bar{X\;}$ (SD)	
Team	220.83 (120.68)		503.33 (165.73)	
Individual	25.83 (7.36)		29.83 (8.50)	
	*n*	%	*n*	%
Service setup				
Integrated	5	83.3	0	0.0
Stand-alone	1	16.7	6	100.0
Staff roles				
Psychiatrists	6	100.0	6	100.0
Nurses	6	100.0	6	100.0
Nurse assistants	2	33.3	1	16.7
Psychologists	6	100.0	6	100.0
Occupational therapists	3	50.0	5	83.3
Social workers	3	50.0	4	66.7
Support workers	3	50.0	5	83.3
Peer support workers	6	100.0	1	16.7
Advocates/volunteers	0	0	1	16.7
Weekly team meetings	6	100.0	6	100.0
Supervision arrangements				
Individual	5	83.0	6	100.0
Group	6	100.0	3	50.0

### Preliminary psychometric properties of the COM-FIDE measure

#### Reliability analysis

Item-level calculations of the G estimate of reliability suggested a potentially good inter-rater reliability across the measure. All but two items showed coefficients above 0.6, and 17 of the 32 items (53,1%) showed coefficients above 0.9 (Table 2). The item ‘Flexibility of Response’ had a reliability coefficient of 0.42 and the item “OD continued professional development” had a coefficient of 0, given its null variance (rate variance = 0.000, rater variance = 0.000, estimated variance of the combination of rate*rater interaction and residual effects = 2.298).

**Table 2:** Inter-rater reliability of the COM-FIDE using the G estimate (*n* = 24).

Item	*G*(0.200, 2)
COM-FIDE scale	0.992
Team structure and culture	
1. Team ethos and comprehensiveness	0.914
2. Staff training	0.868
3. Supervision	0.829
4. Staff roles	0.918
5. Team capacity	0.897
6. Routine outcome monitoring	0.952
7. Safety	0.896
8. Service-user involvement in co-production	0.944
Access and engagement	
1. Access to the service	0.927
2. Providing information	0.689
3. Prompt action	0.818
4. Identification of support systems	0.916
5. Flexibility of response	0.421
6. Assertive engagement	0.913
Delivery of care	
1. Continuity of care	0.896
2. Establishing clinical meetings	0.918
3. Collaborative decision making	0.950
4. Information sharing and communication	0.751
5. Service-user involvement in the delivery of care	0.829
6. Coordination of care	0.646
External support	
1. Service linkage	0.884
2. Community links (Practitioner level)	0.783
3. Community links (Support system)	0.929
4. Caregiver involvement and support	0.969
5. Discharge and aftercare	0.760
Open dialogue addendum	0.997
1. Transparency	0.929
2. Self-disclosure	0.970
3. Intervision frequency	0.990
4. Intervision content and structure	0.995
5. Team self-work	0.964
6. OD training	0.995
7. OD continued professional development	0.000

#### Internal consistency

Both the 25-item COM-FIDE scale and the 7-item OD addendum suggested potentially good internal consistency, with Cronbach’s alpha coefficients of 0.90 on the overall COM-FIDE scale and 0.95 in the OD addendum (see Table 3 for subscale-specific coefficients). An item-level analysis was conducted to examine whether deleting any individual item would make important changes to the overall internal consistency of each scale. Results suggested little influence of any individual item on the total internal consistency of the 25-item COM-FIDE scale (coefficient change ranging from-0.002 to 0.01) and the 7-item OD addendum (range = −0.020–0.016).

**Table 3:** Internal consistency of COM-FIDE subscales.

COM-FIDE subscale (*n* = 24)	Internal consistency (Cronbach’s alpha)
Team structure and culture	0.681
Access and engagement	0.677
Delivery of care	0.817
External support	0.713
Open Dialogue addendum	0.954

When analyzed on a section level, all 5 sections appeared to have adequate internal consistency (Table 3). Results suggested little influence of any individual item on the total internal consistency of their respective section (coefficient increases ranging from 0.02 to 0.04 across sections); however, some items showed very small item-total correlations (minimum value of 0.3; Field, 2017). Further, some items were found to negatively correlate with their sub-samples. For instance, in the ‘Team structure and culture’ section, items ‘Supervision’ had a negative item-total correlation of-0.01 as did ‘Training’ and ‘Staff roles’, with coefficients of-0.25 and-0.29, respectively. Also, in the ‘Access and engagement’ section, item ‘Flexibility of response’ had a negative item-total correlation of-0.04. All other items had item-total correlation coefficients above 0.4.

#### Item scores

On an item level, 6 of the 25 COM-FIDE items (24%) had mean scores equal to or above 3.40 (‘very good’); 14 items (56%) had scores between 2.80 and 3.39 (‘good’); two items (8%) had scores between 2.40 and 2.79 (‘acceptable’); and three items (12%) had scores below 2.39 (‘poor’; Table 4).

**Table 4:** Differences in COM-FIDE mean scores between service models (*n* = 12).

	Open Dialogue (*n* = 6)		Standard care (*n* = 6)	
	Mean (SD)	Range	Mean (SD)	Range
COM-FIDE score	3.25 (0.38)	2.78–3.72	2.97 (0.35)	2.72–3.66
Team structure and culture	3.02 (0.37)	2.56–3.44	2.99 (0.35)	2.63–3.63
Access and engagement	3.26 (0.40)	2.58–3.75	3.15 (0.44)	2.58–3.83
Delivery of care	3.35 (0.51)	2.67–4.00	2.65 (0.48)	2.17–3.50
External support	3.47 (0.34)	3.10–3.90	3.10 (0.44)	2.60–3.70
Open dialogue addendum	3.44 (0.36)	2.93–3.79	1.30 (0.30)	1.00–1.86

### Standard of care (COM-FIDE score)

**Figure 2:**
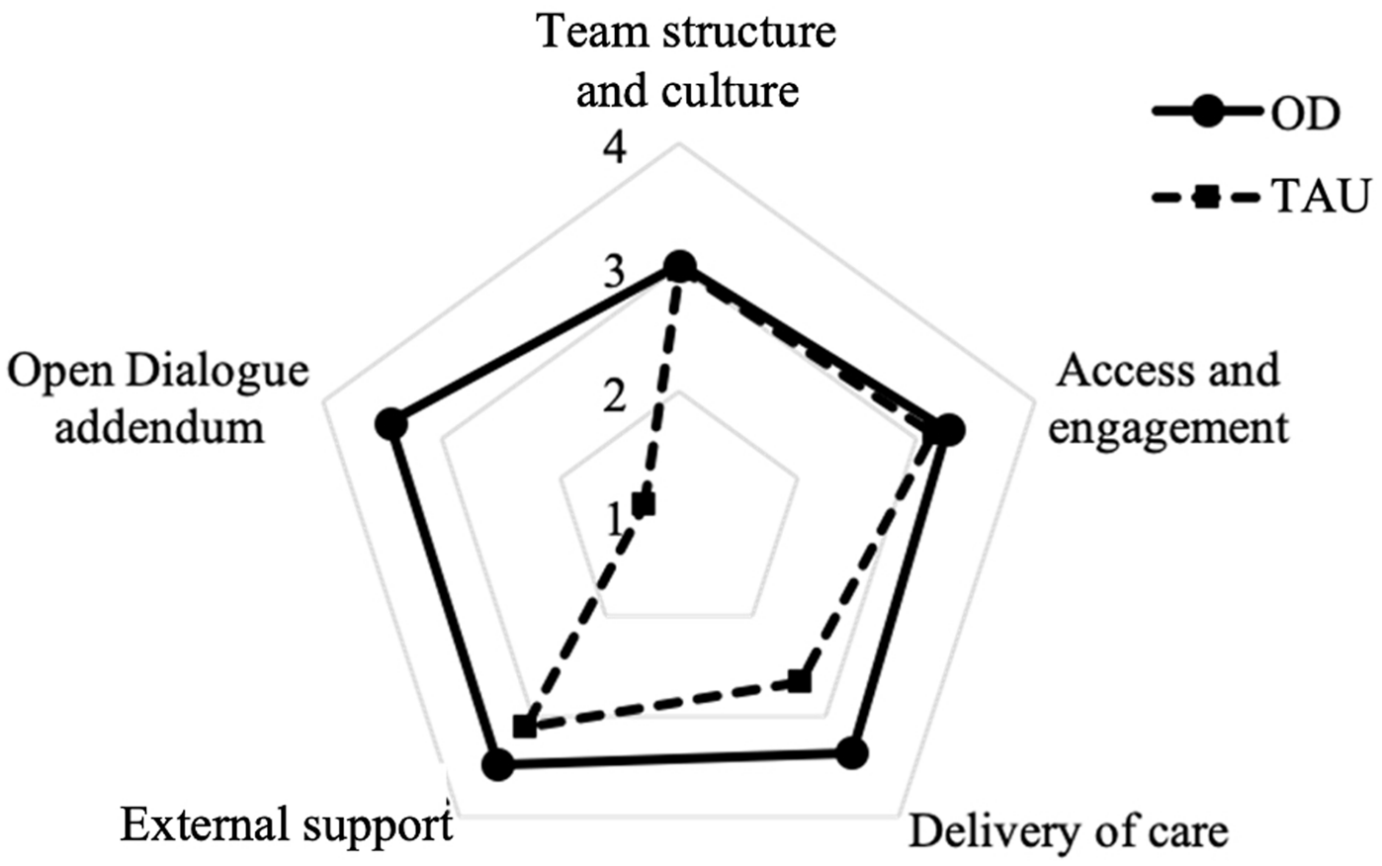
Comparison of mean COM-FIDE section scores between Open Dialogue (OD) and standard care (TAU).

Overall, the mean COM-FIDE total score (i.e., excluding the OD addendum, as this dimension was only relevant to OD teams) across all 12 teams was 3.11 (SD = 0.38, range = 2.72–3.72), possibly suggesting ‘good’ fidelity to standard NHS care. When analyzed by model of care, the 6 OD teams had a mean COM-FIDE total score of 3.25 (SD = 0.38; range = 2.78–3.72), whereas the 6 TAU teams had a mean COM-FIDE total score of 2.97 (SD = 0.35, range = 2.72–3.66). Open dialogue teams had higher scores in all sections compared to TAU teams (Figure 2).

**Figure 3:**
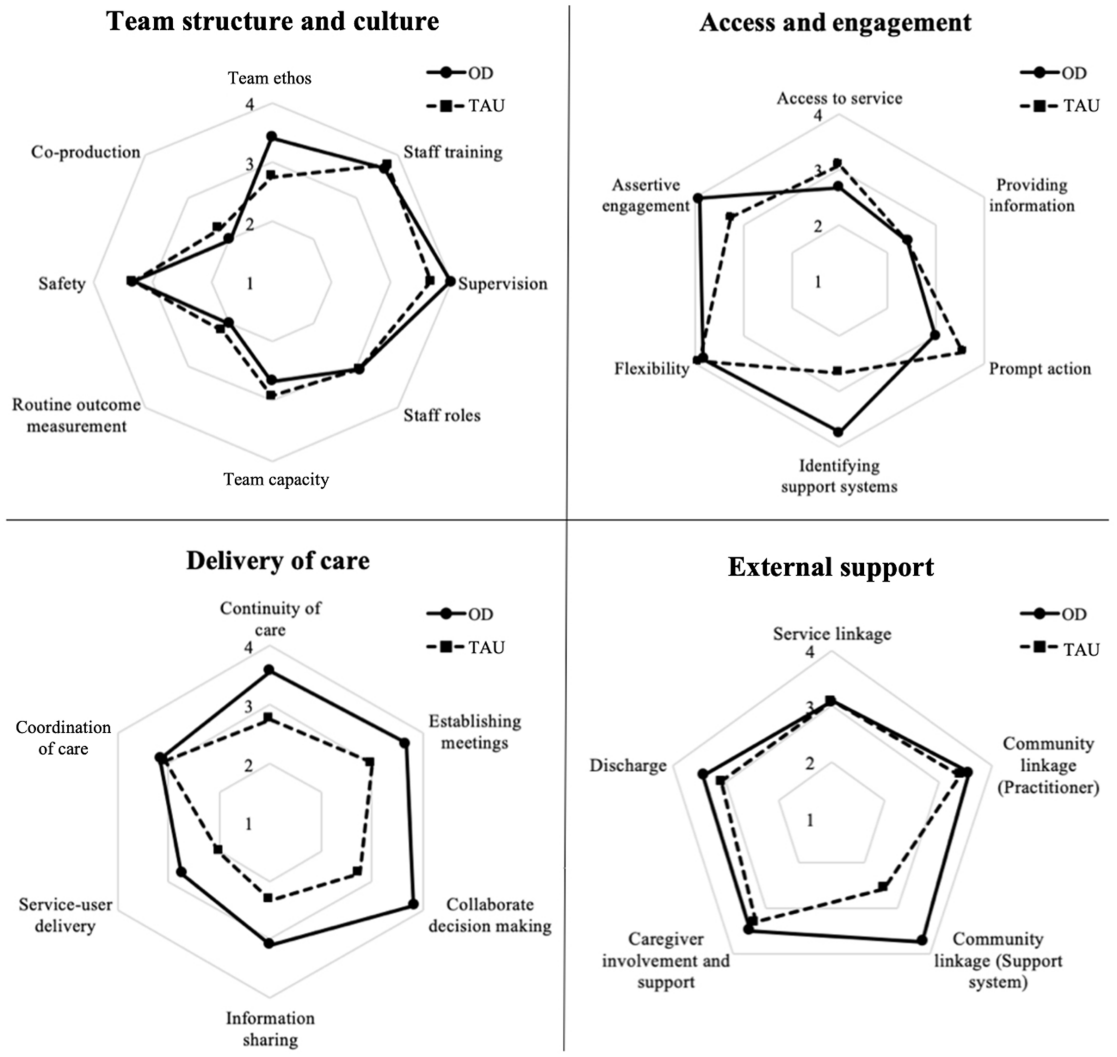
Comparison of mean COM-FIDE item scores between Open Dialogue (OD) and standard care (TAU).

Overall, OD teams scored higher on most items. TAU teams scored higher than OD teams in ‘co-production’ (mean = 2.25, SD = 0.52), ‘service capacity’ (mean = 2.92, SD = 0.49) ‘routine outcome measurement’ (mean = 2.17, SD = 0.26), ‘access to the service’ (mean = 3.08, SD = 0.66), and ‘prompt action’ (mean = 3.58, SD = 0.58; Figure 3).

### Open Dialogue fidelity

**Figure 4:**
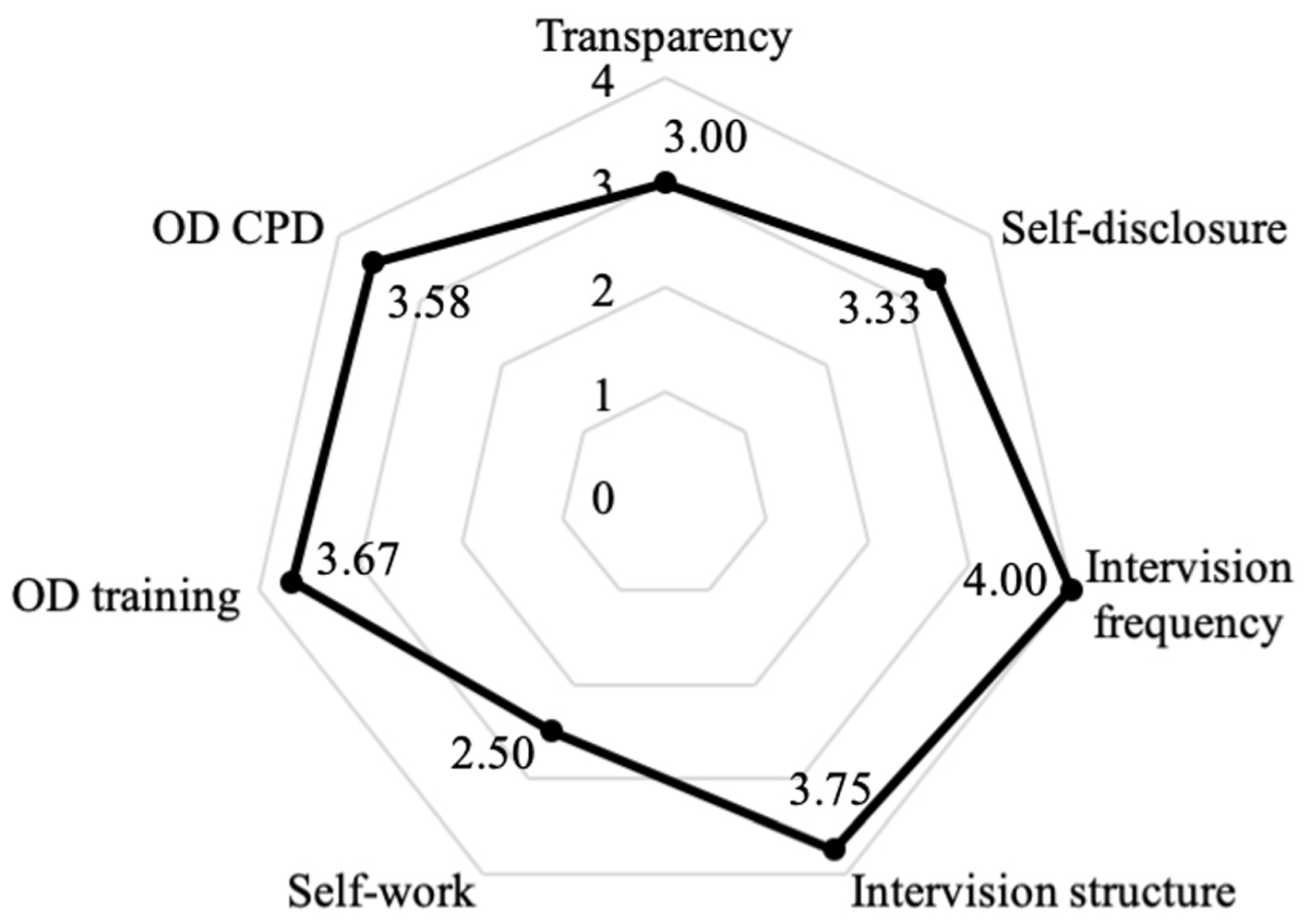
Mean scores of the Open Dialogue (OD) addendum (Open Dialogue teams). CPD=Continued professional development.

When focusing only on the 6 OD teams, three of the 6 teams (50%) showed ‘very good’ fidelity, 2 teams (33%) were in the ‘good’ range, and one team (17%) demonstrated ‘acceptable’ fidelity. On an item level, 4 of the 7 items (57.1%) had mean scores equal to or above 3.40 (‘very good’); two items (14.2%) had scores between 2.80 and 3.39 (‘good); and one item (14.2%) had scores between 2.40 and 2.79 (‘acceptable’; Figure 4).

## Discussion

### The Community Mental Health Team Fidelity Scale

These preliminary findings suggest that COM-FIDE is a robust measure of program fidelity for crisis and continued community care teams aiming at integrating OD to their practice. This is in line with the findings on the CYPRESS measure (Gaffney, 2012), which was shown to be a robust measure for assessing MST fidelity. As noted by Waters et al. (2021) in a recent discussion paper, there are significant commonalities between COM-FIDE general scale and the CYPRESS scale. Both were developed by the same research group and designed to capture all core components of well-functioning community-based teams (in the case of CYPRESS, for services for children and young people). The design of COM-FIDE supports its use as a measure of fidelity for current standard community care (the comparator in many evaluations). In establishing the ODDESSI program, the research group drew a distinction between OD as an organizational intervention (i.e., fidelity) which is measured by the COM-FIDE measure, and a therapeutic intervention (i.e., adherence) which is measured by the OD Adherence Scale (Lotmore et al., 2022). While this approach will require additional reviews we believe this is more than compensated for by allowing for the key organizational elements of OD and standard care to be robustly compared. The adherence measure is only of relevance for OD services.

In terms of reliability, inter-rater reliability is promising especially considering that none of the raters were OD-trained. Although three items of the general scale and one item from the OD addendum were below acceptable ranges, it is possible that this was a consequence of unclear behavioral anchors. Interestingly, both ‘providing information’ and ‘coordination of care’ received mixed feedback from experts. Developers argued that providing information about the service to clients and referrers helps streamline access to the service; however, there were some doubts on whether these two features were too similar to tease apart during interviews. Similarly, coordination of care was considered a key component of crisis and continued community care; however, there were concerns about this item being redundant. With regards to ‘flexibility of response’, the low reliability may have been due to the lack of clarity in the definition, which made it difficult for raters to reach a consensus in scores. As per the lack of inter-rater variance in the OD-specific item of “continued professional development,” this may have been because all OD sites attended the same CPD programe and anchors were not sensitive enough to identify major differences in extended training beyond percentages of staff engagement. Future versions of the manual could include clearer definitions and more specific behavioral anchors.

In terms of validity, COM-FIDE appears to have adequate content validity and the ODDESSI team considered it feasible for use in the full trial. The iterative item refinement process, as well as the discussions with international experts in the field (including the developer of Open Dialogue) were central to developing items that would fit both models of care while also being sufficiently sensitive to possibly distinguish between them.

### Defining a ‘good’ standard of care

Results suggest that all teams demonstrated a ‘good’ standard of care against the criteria set out above. Most OD teams developed from TAU teams (except for one team which was an independent team prior to the trial); with a varying degree of experience, staffing, and capacity across teams.

The four-tier cut-off approach was chosen as a solution based on existing literature on fidelity measurement. Although it proved useful in determining whether participating teams were ready for inclusion in the trial (i.e., ‘acceptable’ fidelity) it was limited in setting variations in fidelity above the cut-off. Further data collected across a range of OD and a range of community mental health teams should support further refinement of the scale.

### Strengths and limitations

COM-FIDE is a feasible and reliable measure for use in the ODDESSI program and is the first measure to explicitly address service level delivery of open dialogue. Its development and results from the present study identified a number of strengths but also highlighted some limitations of the measure.

One of the main strengths of the study is in the measure development process. One of the aims of the ODDESSI trial was to comprehensively assess the organizational and therapeutic elements of OD by developing valid and reliable measures to compare OD versus current standard care. We believe this was best achieved through two distinct measures (i.e., COM-FIDE and OD Adherence Scale). Having the opportunity to discuss and revise the measure with experts in the field allowed for a rich discussion about the theoretical ‘critical components’ of the Finish OD approach to translate the therapeutic principles (Seikkula et al., 1995), and key elements (Olson et al., 2014; Ziedonis et al., 2015) into measurable structural and therapeutic variables. A modified Delphi approach to expert feedback (Dalkey and Helmer, 1963) may have nonetheless provided more structure to the measure development process.

In terms of limitations, a larger sample would have allowed for more robust methods (e.g., factor analysis); but as noted above this could be addressed when additional data becomes available. Another limitation was that raters were not fully crossed or nested given the difficulties in matching respondent and rater availability. This limitation was addressed in two ways: first, the G estimator–although unconventional–seemed a robust solution to this as it considers rater assortment and systematic rater deviations; and as interviews were recorded it is possible to further assess reliability using novel independent raters.

## Conclusion

This paper describes the development, piloting, and testing of a program fidelity measure for its use in the ODDESSI program. The Community Mental Health Team Fidelity Scale (COM-FIDE) captures both standard NHS crisis care practice and open dialogue practice. The measure development process used recognized methods including multiple raters, multiple data sources, and multiple settings to assess its properties. Preliminary psychometric results were encouraging, suggesting that COM-FIDE is suitable for use in a range of community mental health settings. Results suggest that COM-FIDE may be able to establish (a) the extent to which teams deliver their respective models according to their protocols, and (b) the degree of differentiation between similar approaches to crisis care and recovery.

## Data availability statement

The raw data supporting the conclusions of this article will be made available by the authors, without undue reservation.

## Ethics statement

The studies involving human participants were reviewed and approved by this study received ethics approval from the Health Research Authority under reference number 18/LO/0026. No personal or confidential information was solicited. Participants gave consent for being recorded using an encrypted and password-protected recorder. The patients/participants provided their written informed consent to participate in this study.

## Author contributions

MA-M, ML, and SP contributed to conception and design of the study. MA-M organized the database, performed the statistical analysis, and wrote the first draft of the manuscript. All authors contributed to data collection, manuscript revision, read, and approved the submitted version.

## Funding

This work was supported by the National Institute for Health Research (NIHR) under grant RP-PG-0615-20021, the NIHR UCLH Biomedical Research Center, and the Mexican National Council for Science and Technology (CONACYT) under scholarship 600356. The views presented are those of the authors and may not reflect those of the funding agencies who did not have input into the manuscript.

## Conflict of interest

The authors declare that the research was conducted in the absence of any commercial or financial relationships that could be construed as a potential conflict of interest.

The reviewer TB declared a past co-authorship with the author MA-M to the handling editor.

## Publisher’s note

All claims expressed in this article are solely those of the authors and do not necessarily represent those of their affiliated organizations, or those of the publisher, the editors and the reviewers. Any product that may be evaluated in this article, or claim that may be made by its manufacturer, is not guaranteed or endorsed by the publisher.
